# Molecular Crosstalk between Integrins and Cadherins: Do Reactive Oxygen Species Set the Talk?

**DOI:** 10.1155/2012/807682

**Published:** 2011-12-13

**Authors:** Luca Goitre, Barbara Pergolizzi, Elisa Ferro, Lorenza Trabalzini, Saverio Francesco Retta

**Affiliations:** ^1^Department of Clinical and Biological Sciences, University of Torino, 10043 Orbassano, Italy; ^2^Department of Biotechnology, University of Siena, 53100 Siena, Italy

## Abstract

The coordinate modulation of the cellular functions of cadherins and integrins plays an essential role in fundamental physiological and pathological processes, including morphogenesis, tissue differentiation and renewal, wound healing, immune surveillance, inflammatory response, tumor progression, and metastasis. However, the molecular mechanisms underlying the fine-tuned functional communication between cadherins and integrins are still elusive. This paper focuses on recent findings towards the involvement of reactive oxygen species (ROS) in the regulation of cell adhesion and signal transduction functions of integrins and cadherins, pointing to ROS as emerging strong candidates for modulating the molecular crosstalk between cell-matrix and cell-cell adhesion receptors.

## 1. Introduction

The communication between signaling pathways, the so-called molecular crosstalk, plays a central role in cell biology, enabling the cell to couple the molecular functions of either near neighbors or distant cell components, with resulting synergistic or antagonistic effects and eventually appropriate biological outcomes.

Among the most important cellular crosstalk events is the signaling network that couples the molecular functions of adhesion receptors of the integrin and cadherin families. Indeed, acting in concert with growth factor receptor signaling pathways, this regulatory network is fundamentally important during the entire life of all metazoans, whereas its dysfunction almost invariably leads to developmental defects and/or diseases, including genetic diseases and cancer [1].

Integrins and cadherins are the major cell-extracellular matrix (ECM) and cell-cell adhesion receptors, respectively, and represent critical determinants of tissue architecture and function both in developing and adult organisms [2, 3].

Integrins are heterodimeric transmembrane glycoproteins composed of noncovalently linked *α* and *β* subunits, which are endowed with both structural and regulatory functions. They link the ECM to several distinct cytoplasmic proteins and the actin cytoskeleton at focal adhesions, thus serving as organizing centers for the assembly of structural and regulatory protein complexes at discrete cell-matrix adhesion sites and providing a mechanically sensitive system for mechanotransduction [4]. Furthermore, often acting in concert with growth factor receptors, they provide both outside-in and inside-out transmission of signals across the plasma membrane that control a number of critical cellular processes, including adhesion, cytoskeleton remodeling, migration, proliferation, differentiation, apoptosis, and gene expression [2, 5]. Specifically, integrin-mediated outside-in signaling stimulates tyrosine phosphorylation and activation of several proteins, including major components of focal adhesions, such as Src and FAK nonreceptor tyrosine kinases (PTK), and paxillin, as well as receptor tyrosine kinases (RPTK). In turn, these proteins are antagonized by nonreceptor (PTP) and receptor tyrosine phosphatases (RPTP), and entwined in a dynamic interplay with small GTPases and components of specialized plasma membrane and endosome microdomains, including caveolin-1, to form compartmentalized signaling platforms that allow for temporal and spatial coordination of specific downstream signaling events [6, 7].

Cadherins are single-pass transmembrane glycoproteins that support calcium-dependent, homophilic cell-cell adhesion. Together with their cytoplasmic domain interactors, such as *β*-catenin and p120^ctn^, they constitute the core components of adherens junctions (AJs). These specialized adhesive structures link the cadherin homophilic adhesion to the actin cytoskeleton and are required for formation and maintenance of stable cell-cell adhesion and differentiated phenotype in all solid tissues [3, 8, 9]. Cadherin endocytosis and endosome-mediated trafficking has emerged as a major mechanism for controlling AJ remodeling [10–17]. Moreover, growing evidence demonstrates that cadherins can modulate the signaling activity of several proteins, including *β*-catenin, Ras and Rho family GTPases, PTK, RPTK, PTP, and RPTP, as well as mechanotransduction pathways that affect membrane and actin cytoskeleton dynamics [3, 18–21].

Although there is a large body of evidence supporting the existence of a fine-tuned crosstalk between members of these two adhesive receptor families, which influences their expression, turnover, positioning, and/or functions, and may enhance or suppress adhesion depending on the cellular and environmental context [1, 10, 17, 22–41], the molecules and molecular mechanisms involved in such important phenomenon are not completely defined. To clarify how this crosstalk is regulated remains therefore a fundamental challenge for basic and translational research, including research on tumor and vascular disease progression.

Multiple molecules and regulatory mechanisms have been placed at the heart of the molecular crosstalk between integrins and cadherins, including small GTPases of the Ras and the Rho families [10, 17, 42–45], nonreceptor kinases such as Src, FAK, Fer, and PI3K [27, 34, 46, 47], cell surface receptor-mediated pathways [48–50], and adhesion-dependent actomyosin traction forces [26, 34, 51].

Previously, we reported the pivotal role of the small GTPase Rap1 in regulating the crosstalk between cadherins and integrins, suggesting a model where Rap1 acts as a turnabout for endosome signaling and membrane trafficking pathways to orchestrate the delivery of cadherins and integrins to specific cell-cell and cell-matrix landmarks at the plasma membrane, respectively [10, 17].

Intriguingly, recent growing evidence suggests that reactive oxygen species (ROS) play an important role in both integrins, small GTPases, and cadherins functions, raising the possibility that ROS may contribute to the modulation of the molecular crosstalk between integrins and cadherins.

In this paper, we discuss the most recent advances on the role of ROS in outside-in and inside-out signal transduction events implicating integrins and cadherins, providing building bloks for the hypothesis that ROS constitute important players in the molecular crosstalk between these cell adhesion receptors.

## 2. ROS Metabolism and Signaling

ROS are a highly reactive group of oxygen-containing molecules, including free radicals and peroxides, such as superoxide anion (O_2_^∙−^) and hydrogen peroxide (H_2_O_2_), which are generated constitutively, as common by-products of oxidative metabolism, or in response to the activation of several oxidative enzyme complexes [52–55].

**Figure 1:**
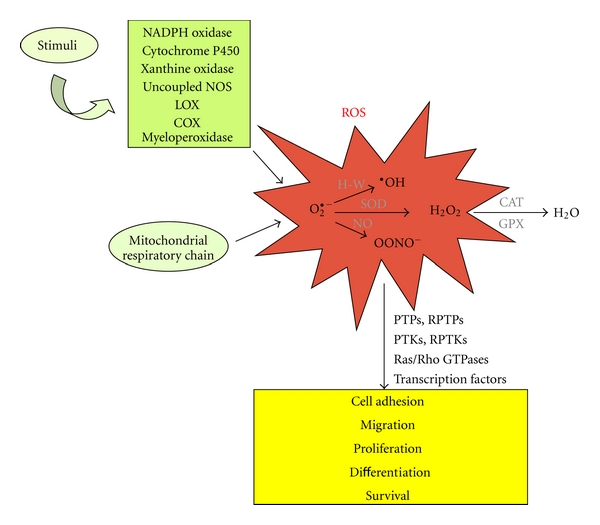
Schematic representation of ROS metabolism and signaling. The superoxide anion (O_2_^∙−^) is a key determinant of oxidative effects as well as the precursor of all other major reactive oxygen species, including hydroxyl radical (^∙^OH), hydrogen peroxide (H_2_O_2_), and peroxynitrite (OONO^−^). It is generated constitutively as by-product of oxidative metabolism, as well as upon stimuli triggering the activation of oxidative enzymes, including NADPH oxidases, xanthine oxidases, cytochrome P450 monooxygenases, uncoupled NO synthase (NOS), myeloperoxidases, lipoxygenases (LOX), and cyclooxygenases (COX). Conversely, O_2_^∙−^ is removed by superoxide dismutase (SOD) enzymes, which catalyze the dismutation of O_2_^∙−^ into H_2_O_2_ and O_2_. In turn, H_2_O_2_ is reduced to H_2_O by the catalase (CAT) and glutathione peroxidase (GPX) enzymes. At physiologic concentrations, ROS are endowed with essential signaling properties, being involved in the redox-dependent regulation of multiple signal transduction pathways to fulfill a wide range of essential biological processes, including cell adhesion, migration, proliferation, differentiation, and survival. However, at high levels, ROS exert very damaging effects through oxidative stress. H-W: Haber-Weiss reaction; NO: nitric oxide.

The superoxide anion (O_2_^∙−^) is the key determinant of the overall effects of ROS. Indeed, even though it has a short half-life, O_2_^∙−^ is the precursor of all other major reactive oxygen species found in biological systems, including the powerful oxidants hydroxyl radical (^∙^OH), hydrogen peroxide (H_2_O_2_), and peroxynitrite (OONO^−^) [52–55]. It is generated by a number of sources located throughout the cell via the incomplete, one-electron reduction of molecular oxygen (O_2_). Specifically, under physiological conditions the redox complexes I (NADH/ubiquinone oxidoreductase) and III (ubiquinol/cytochrome c oxidoreductase) of the mitochondrial electron transport chain are the major constitutive source, converting up to 5% of molecular O_2_ to O_2_^∙−^ [56]. In addition, O_2_^∙−^ is produced by the activity of NAD(P)H oxidases, xanthine oxidases, cytochrome P450 monooxygenases, uncoupled NO synthase (NOS), myeloperoxidases, lipoxygenases, and cyclooxygenases [52–55], which can be induced by a variety of chemical and physical stimuli, including integrin ligands, growth factors, G-protein coupled receptor agonists, cytokines, neurotransmitters, metabolic factors, shear stress, ischemia/reperfusion, chemotherapeutics, and ionizing radiations, as well as aging [52–54, 57, 58]. Conversely, O_2_^∙−^ is rapidly removed by distinct superoxide dismutase (SOD) isoenzymes, located in the mitochondria (SOD2), cytoplasm (SOD1), and extracellular (SOD3) compartments, which catalyze the dismutation of O_2_^∙−^ into H_2_O_2_ and O_2_. In turn, H_2_O_2_ is reduced to H_2_O by the catalase and glutathione peroxidase enzymes. In addition, O_2_^∙−^ can be converted to hydroxyl radical (^∙^OH) by the Fenton or Haber-Weiss reactions, or to peroxynitrite (OONO^−^) by reacting with nitric oxide (NO) [59] (Figure 1).

 It is now well established that physiologic concentrations of ROS are endowed with essential signaling properties, which are mainly due to the reversible oxidation of redox-sensitive molecular targets, thereby functioning as signaling molecules. Accordingly, it has been clearly demonstrated that ROS are involved in the redox-dependent regulation of multiple signal transduction pathways to fulfill a wide range of essential biological processes, including cell adhesion, migration, proliferation, differentiation, and survival [52–55]. However, at high levels, ROS are known to exert very damaging effects through oxidative stress. This is caused by an imbalance between the production of ROS and the ability of cellular antioxidant mechanisms to readily detoxify the reactive intermediates. Importantly, because O_2_^∙−^ can spontaneously react with NO to form OONO^−^ at a rate 3 times faster than O_2_^∙−^ dismutation by SOD, modest increases of O_2_^∙−^ can result in a great reduction of NO bioavailability and increased formation of OONO^−^, a very strong oxidant with the potential to produce multiple cytotoxic effects [60, 61]. In addition, OONO^−^ can also trigger feedforward mechanisms that further amplify O_2_^∙−^ generation and oxidative stress, including the uncoupling of NO synthase (NOS) which produces O_2_^∙−^ instead of NO, thus amplifying the risk of cellular dysfunction and oxidative injury [52]. The maintenance of highly regulated mechanisms to control ROS levels and functional specificity is therefore essential for normal cellular homeostasis and proper response to environmental stimuli.

Among the major source of ROS, NADPH oxidases have been demonstrated to play a fundamental role in the compartmentalization of ROS production and redox signaling [7].

The NADPH oxidase (NOX) complex was originally identified in phagocytic leukocytes as an enzymatic defense system against infections required for the oxidative burst-dependent microbial killing [62, 63]. It is composed of membrane-associated and cytosolic components, which assembly to form the active NOX enzymatic complex in response to appropriate stimuli. Specifically, this complex consists of membrane-associated cytochrome b558, comprising the catalytic gp91^phox^ (also known as NOX2) and regulatory p22^phox^ subunits, and four cytosolic regulatory components, including p40^phox^, p47^phox^, p67^phox^, and the small GTPase Rac1 [63]. Subsequently, NADPH oxidase complexes were also found in nonphagocytic cells, where several isoforms of the catalytic NOX2 protein were identified, including NOX1, NOX3, NOX4, and NOX5, and shown to localize in proximity of specific redox-sensitive molecular targets within discrete subcellular compartments, thereby facilitating the compartmentalization of redox signaling [7]. Indeed, NADPH oxidases can be targeted and activated within caveolae/lipid rafts, focal adhesions, cell-cell contacts, lamellipodial leading edges and membrane ruffles, endosomes, and the nucleus, allowing spatiotemporally confined ROS production and activation of specific redox signaling events [7].

Besides NADPH oxidase, an important role in the spatio-temporal regulation of ROS production is also played by enzymes involved in arachidonic acid (AA) metabolism, such as phospholipase A_2_(PLA_2_), lipooxygenases (LOX), and cyclooxygenases (COX), suggesting that a complex regulatory network may take place for proper modulation of redox signaling [64]. 

Accumulating evidence points to PTPs as the major redox-sensitive molecular targets of ROS [65]. This protein family is indeed characterized by the presence in the active site of a highly conserved sequence motif containing a Cys residue that is essential for catalysis and very susceptible to reversible inactivating oxidation by ROS. In turn, oxidative inactivation of PTPs promotes phosphorylation-dependent downstream signaling events. In addition to PTPs, other important signaling proteins have been shown to act as endogenous redox sensors for mediating ROS signaling, including RPTKs, cytoplasmic kinases, small GTPases of the Ras and Rho families, and transcription factors [7, 65, 66] (Figure 1). Conversely, protein oxidation can be reversed by thiol donors such as glutathione [67]. 

Remarkably, the activation of redox signaling complexes at integrin-mediated cell-matrix adhesion sites and cadherin-mediated cell-cell junctions induces opposite effects, leading to the assembly of focal adhesions and the disassembly of adherens junctions, respectively [7].

## 3. ROS and Integrins

**Figure 2:**
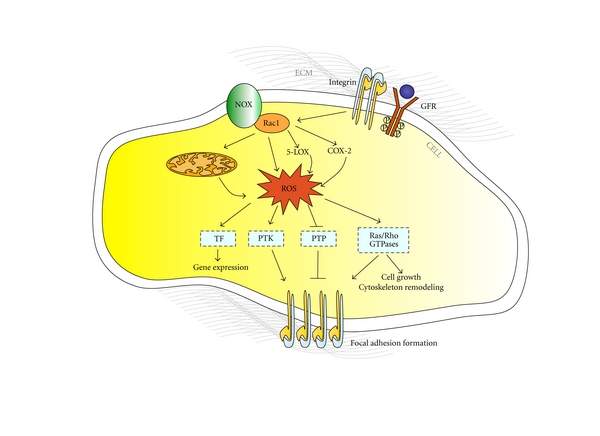
ROS mediate integrin outside-in signaling. Integrin engagement with extracellular matrix (ECM) proteins triggers a transient and localized burst of ROS, either independently or in cooperation with growth factor receptors (GFR), which is essential to the proper transduction of outside-in signaling pathways. The small GTPase Rac1 acts as a crucial upstream regulator of ROS production, orchestrating integrin outside-in signaling-mediated changes in mitochondrial metabolic/redox function, and activation of distinct oxidases, including NADPH-oxidases (NOX), 5-lipoxygenase (5-LOX), and cyclooxygenase-2 (COX-2). The signaling properties of integrin-induced ROS are largely due to the reversible oxidation of specific subsets of redox-sensitive proteins, including oxidative inhibition of PTPs, and activation of PTKs, RPTKs, small GTPases of the Ras and Rho families, and transcription factors (TF) such as AP-1 and NF-*κ*B.

It is now well established that ROS are implicated in regulating many integrin-mediated cellular responses, including adhesion, cytoskeleton organization, migration, proliferation, differentiation, and survival. Indeed, a large body of evidence demonstrates that integrin activation triggers a transient and localized burst of ROS, either independently or in cooperation with growth factor receptors, which is essential to the proper transduction of outside-in signaling pathways [7, 47, 68]. Specifically, although the underlying molecular mechanisms remain to be precisely defined, there is clear evidence that integrin engagement with antibodies or extracellular matrix proteins triggers ROS production by promoting changes in mitochondrial metabolic/redox function [69–71], and activation of distinct oxidases, including NADPH-oxidases [47, 72, 73], and the AA-metabolizing enzymes 5-lipoxygenase (5-LOX) [70, 72] and cyclooxygenase-2 (COX-2) [74]. Conversely, there is evidence that ROS can also influence integrin-mediated inside-out signaling by inducing the conformational change required for integrin activation [64]. Remarkably, the small GTPase Rac1 has emerged as a crucial, common upstream mediator of ROS production in integrin-mediated outside-in signaling [64, 69–72]. Consistently, Rac1 acts upstream of both NADPH oxidase [7] and AA-metabolizing enzymes, such as PLA_2_ [75, 76], 5-LOX [70, 72, 76], and COX-2 [77], whereas many reports show that AA metabolism modulates NADPH oxidase and mitochondrial ROS production, as well as the existence of a bidirectional signaling crosstalk between mitochondria, and NADPH oxidase, suggesting that Rac1 can orchestrate a complex web of regulation for ROS production [64, 78] (Figure 2). In addition, it is becoming evident that the formation of focal adhesions promotes the assembly of redox signaling platforms, involving integrins, growth factor receptors, and NADPH oxidases, which are essential for localized ROS production and activation of specific redox signaling pathways that mediate adhesion-dependent cell functions [7]. Furthermore, there is evidence that fine-tuned sequential compartmentalization and kinetics of ROS production can account for the modulation of distinct subsets of redox-sensitive signaling molecules involved in early and late phases of cell adhesion, leading to distinct outcomes [70, 79].

 The signaling properties of integrin-induced ROS are largely due to the reversible oxidation of target proteins, and especially of PTPs, as the activity of these proteins is dependent on reactive cysteine residues (Cys-SH) at their active site that are readily susceptible to reversible oxidation [7, 65]. Indeed, ROS produced locally by the synergistic action of integrins and growth factor receptors on NADPH oxidase, as well as on mitochondria and 5-LOX, have been shown to induce oxidative inactivation of distinct PTPs, including the low-molecular-weight protein tyrosine phosphatase (LMW-PTP), PTP1B, PTEN, and SHP2, preventing these enzymes from dephosphorylating and inactivating specific targets, and thereby promoting downstream adhesion-related signaling events (Figure 2). Consistently, integrin-mediated adhesive and signaling functions are significantly mimicked by PTP inhibition [80]. However, ROS generated by integrin activation can also activate PTKs and RPTKs through either direct oxidation of susceptible cysteine residues or indirect inhibition of negative regulatory PTPs [68], whereas the synergistic cooperation between integrins and growth factor receptors expands enormously the plethora of ROS-regulated target proteins to include redox-sensitive small GTPases of the Ras superfamily and transcription factors such as AP-1 and NF-*κ*B [7, 81–83] (Figure 2).

Remarkably, ROS production has often a dual role in small GTPase regulation, leading to either inhibition or activation under different conditions [83–88]. In particular, the inactivation of RhoA has been shown to occur indirectly through a signaling cascade involving the Rac-stimulated release of O_2_^∙−^ from NADPH oxidase, which in turn inhibits LMW-PTP. Because p190Rho-GTPase-activating protein (p190RhoGAP) is a substrate of LMW-PTP, inactivation of LMW-PTP results in accumulation of the active phosphorylated form of p190RhoGAP, which stimulates the hydrolysis of bound GTP to produce inactive GDP-bound RhoA, thereby determining well-characterized readouts, including decreased cell contractility and stabilization of cell-cell junctions [85, 88]. Conversely, RhoA can be directly activated by ROS-mediated reversible oxidation of two critical cysteine residues located in a unique redox-sensitive motif within the phosphoryl binding loop, leading to characteristic outcomes, including stress fiber and focal adhesion formation and cell-cell junction weakening [84, 89, 90]. On the other hand, there is evidence that ROS can activate Rap1 [91], whose signaling is in turn required for suppression of Ras-generated ROS and protection against oxidative stress and consequent cell dysfunctions [92–94].

## 4. ROS and Cadherins

**Figure 3:**
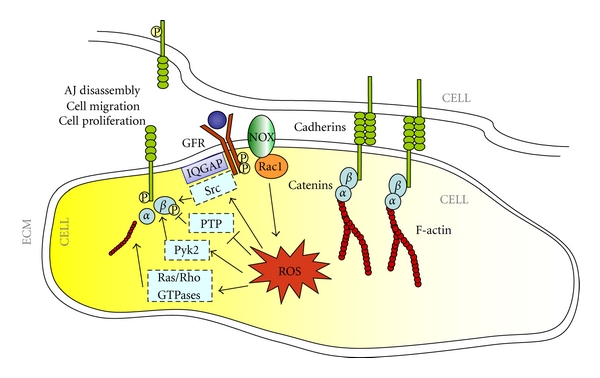
ROS modulate cadherin-mediated cell-cell junctions. Rac1-induced ROS may function as signaling molecules to disrupt cadherin-based cell-cell adhesion through either inhibition or activation of regulatory tyrosine phosphatases and kinases, respectively, as well as by localized activation of IQGAP and small GTPases, leading to various biological responses, including cell migration and proliferation.

Growing evidence demonstrates that ROS play a major role in either stabilization or destabilization of cell-cell junctions mediated by distinct cadherins, including E-, N-, and VE-cadherin [81, 95–99]. In particular, it has been reported that Rac1-mediated ROS production is an essential component in signaling cascades that promote p190RhoGAP translocation to the AJs and the consequent inhibition of local RhoA activity, thus favoring the stabilization of cell-cell contacts [99]. Conversely, clear evidence shows that Rac1-induced ROS function as signaling molecules to disrupt VE-cadherin-based cell-cell adhesion leading to various biological responses, including endothelial barrier dysfunction, enhanced microvascular permeability, and endothelial migration and proliferation involved in angiogenesis [7, 96, 98, 100, 101] (Figure 3). Intriguingly, the apparent contrast between the positive and negative roles of ROS in the maintenance of cadherin-mediated cell-cell junctions correlates with similar features of small GTPases involved in this process, including Rac1, RhoA, and Rap1. Indeed, depending on the extracellular and intracellular context, the activities of Rac1, RhoA, and Rap1 may be not only involved in regulating AJs and endothelial barrier maintenance, but also in active enforcement or disruption of AJs and endothelial barrier integrity, suggesting that the location and duration of the activities of these small GTPases affect the choice of downstream targets, thereby determining distinct biological outcomes [81]. Indeed, while under basal conditions Rac1 enforces the junctions that form the endothelial barrier by promoting ROS-mediated p190RhoGAP recruitment to AJs and the consequent inhibition of local RhoA activity, upon certain growth factor stimuli, including VEGF, it becomes part of a barrier-disturbing mechanism by inducing ROS-mediated phosphorylation of VE-cadherin at Tyr^658^ and Tyr^731^, and *β*-catenin at Tyr^654^, which lead to the disassembly of AJs [81, 96]. Whether ROS act directly on growth factor receptor kinase activity or, more likely, inhibit VE-cadherin-associated tyrosine phosphatases has still to be clarified. In addition, there is evidence for the involvement of the Pyk2 and Src redox-sensitive kinases in the phosphorylation of AJ proteins, including *β*-catenin and p120^ctn^, and the resulting loss of cell-cell adhesion mediated by the Rac1-ROS signaling pathway [95, 100, 102] (Figure 3). Notably, it has been reported that antioxidant compounds can inhibit VEGF-induced angiogenesis through disruption of ROS-dependent Src kinase activation and the subsequent VE-cadherin tyrosine phosphorylation, resulting in the retention of VE-cadherin at cell-cell contacts [100]. Conversely, the cell-cell contact-dependent inhibition of cell growth and stimulation of PTP activity [103] have been associated with a decrease in the steady-state levels of intracellular ROS and the consequent impairment of redox signaling mediated by growth factor receptors [104].

Another component of the Rac1-ROS signaling pathway that plays an important role in the regulation of cadherin adhesive functions is IQGAP, a scaffold protein involved in cellular motility and morphogenesis [105]. IQGAP has been shown to be required for the establishment of VE-cadherin-based cell-cell contacts, and to colocalize and form a complex with VE-cadherin at cell-cell contact sites in quiescent endothelial cells [105]. It may act as a downstream effector of Rac1, as well as an inhibitor of its GTPase activity through a RasGAP-related domain [106–108]. Furthermore, it can facilitate localized ROS production through compartmentalization of Nox2 [109]. Indeed, there is evidence that IQGAP1 plays an essential role in VEGF-stimulated ROS production and VEGFR2-mediated endothelial cell migration and proliferation, suggesting that IQGAP1 may function as a scaffold protein to link VEGFR2 to the VE-cadherin/*β*-catenin complex at AJs, thereby promoting VEGF-stimulated ROS-dependent tyrosine phosphorylation of VE-cadherin, which may contribute to AJ weakening and angiogenesis [105, 110] (Figure 3).

Besides biochemical modification of AJ molecules, the ROS-dependent regulation of cadherins may be also driven by epigenetic events, as a ROS-induced hypermethylation of E-cadherin promoter, due to the upregulation of the transcriptional factor Snail and the recruitment of the DNA methyltransferase-1, and the consequent downregulation of cadherin expression have been reported [111]. 

## 5. ROS as Potential Pivotal Players in the Crosstalk between Integrins and Cadherins

A number of experimental reports have shown that the engagement of integrins with ECM proteins can affect cadherin-containing adherens junctions via multiple mechanisms, including the activation of signaling pathways mediated by small GTPases [10, 17, 42–45], cell surface receptors [48–50], and nonreceptor kinases [22, 27, 34, 47, 112], and the modulation of the actin network [26, 34, 51, 112]. Conversely, there are relatively fewer examples where cadherins have been shown to regulate integrin function [40, 113], but this may be due to the fact that crosstalk in this direction has been explored less extensively. In this context, we have previously reported that the small GTPase Rap1 plays a pivotal role in regulating the crosstalk between cadherins and integrins, suggesting a model where Rap1 acts as a turnabout for endosome signaling and membrane trafficking pathways to orchestrate the control of cadherin and integrin adhesive and signaling functions [10, 17].

**Figure 4:**
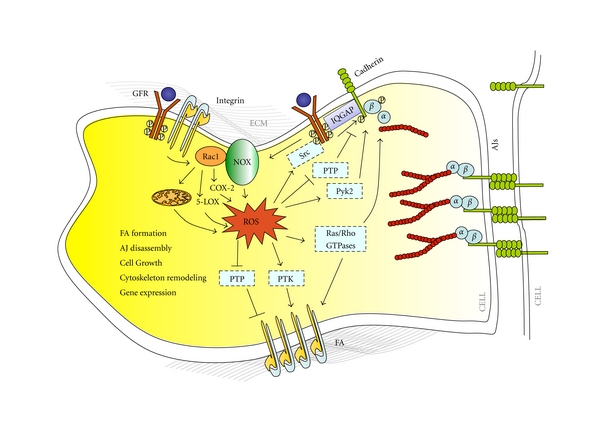
ROS in the crosstalk between integrins and cadherins. ROS generated by integrin activation may influence cadherin adhesive functions by various mechanisms, including inhibition of PTPs and/or activation of PTKs, RPTKs, and IQGAP acting at adherens junctions, as well as spatiotemporal modulation of the activity of redox-sensitive small GTPases and signaling endosomes.

Intriguingly, despite the lack of direct experimental evidence, the large number of studies implicating ROS as major modulators of integrin and cadherin adhesive and signaling functions strikingly supports the thought-provoking hypothesis that ROS play a crucial role in the crosstalk between integrins and cadherins (Figure 4). In particular, there is clear evidence that the assembly of integrin-mediated focal adhesions and the disassembly of cadherin-mediated adherens junctions require the activation of redox signaling complexes involving common regulatory proteins and mechanisms, including redox-sensitive small GTPases and the oxidative inactivation of PTPs [7]. Consistently, both focal adhesions assembly and adherens junctions disassembly are significantly mimicked by oxidative inhibitors of PTPs [10, 80], and prevented by ROS scavengers [95, 96].

 Remarkably, both integrin- and cadherin-related redox signaling pathways involve Rac1 as a key mediator, which is in turn implicated in intimately intertwined functional relationships with other small GTPases, including Ras, RhoA, and Rap1 [7, 64, 69–72, 96, 98, 100, 101, 114].

Furthermore and importantly, recent evidence shows that Rap1 activation by Epac1, a Rap1-GEF involved in the Rap1-dependent regulation of cadherins, may be stimulated by ROS and inhibited by ROS scavengers, indicating that ROS production can trigger Rap1 activation [91]. Conversely, Rap1 signaling has been shown to be required for suppression of Ras-generated ROS and protection against oxidative stress and consequent cell dysfunctions [92–94]. Taking together, these data suggest that the role of Rap1 as pivotal regulator in the crosstalk between cadherins and integrins [10, 17] may underlie feedback mechanisms involving spatially and temporally regulated ROS production and scavenging. Consistently, KRIT1, a Rap1 effector whose loss-of-function mutations are implicated in endothelial cell-cell junction dysfunction and enhanced microvascular permeability underlying the Cerebral Cavernous Malformation disease, has been recently shown to play a role in molecular mechanisms involved in the maintenance of the intracellular ROS homeostasis to prevent oxidative cellular damage [115].

Finally, ROS generated by integrin activation could influence cadherin adhesive functions through the activation of either PTKs and RPTKs, including Src and growth factor receptors [68], or IQGAP, a component of the Rac1-ROS signaling pathway implicated in the modulation of AJ dynamics [105, 110] as well as in signaling downstream from both integrins and RPTKs [116], suggesting a further crosstalk mechanisms (Figure 4).

## 6. Concluding Remarks

It is well established that, besides their structural roles, both integrins and cadherins can provide bidirectional transmission of signals across topographically discrete regions of the plasma membrane. In addition, there is growing evidence supporting the existence of a fine-tuned, bidirectional crosstalk between these adhesion molecules, which may enhance or suppress their adhesive and signaling functions depending on the cellular and environmental context. Indeed, the integrin-cadherin crosstalk is involved in the epithelial-mesenchymal transition (EMT) underlying fundamental physiological and pathological processes, including embryonic development and cancer [22, 25–27, 33, 39].

This paper highlights recent growing evidence supporting a major role of reactive oxygen species (ROS) in both outside-in and inside-out signaling of integrins and cadherins, raising the possibility that ROS constitute master regulators of the crosstalk between these fundamental cell adhesion receptors.

Indeed, over the past few years, it has clearly emerged that outside-in integrin signaling triggers ROS production by several distinct mechanisms, such as changes in mitochondrial metabolic/redox function [69–71] and activation of distinct oxidases, including NADPH oxidase [47, 70, 72–74]. On the other hand, growing evidence demonstrates that ROS play a major role in the regulation of cadherin adhesive and signaling functions by mechanisms involving either biochemical modifications (e.g., phosphorylation) of AJ proteins, including cadherins and catenins, epigenetic modifications of the cadherin promoter, or modulation of small GTPases regulating cadherin-dependent cell-cell adhesion [7, 81, 95–101].

Remarkably, whereas emerging data show that integrin and cadherin redox signaling involves shared regulatory proteins, accumulating evidence suggests that discrete subcellular compartmentalization of ROS constitutes a major mechanism of localizing activation of downstream redox signaling events, thereby playing a critical role in transmitting cell signals in response to various environmental stimuli to regulate distinct cell functions, including cell-matrix and cell-cell adhesion [7, 117]. In particular, ROS production may be localized through interactions of NADPH oxidase with signaling platforms associated with lipid rafts and caveolae, as well as with endosomes [7, 118]. Furthermore, there is evidence that growth factor receptors mediate signaling through a subset of signaling endosomes termed redoxosomes (redox-active endosomes), which are uniquely equipped with redox-processing proteins capable of transmitting ROS signals from the endosome interior to redox-sensitive effectors on the endosomal surface, thereby controlling redox-dependent effector functions through the spatial and temporal regulation of ROS as second messengers [117].

Taken together with the well-established roles of growth factor receptors, small GTPases and endosome signaling in the functional relationship between integrins and cadherins [17], the experimental evidence and observation discussed in this paper point to a novel hypothetical mechanism whereby the spatial and temporal regulation of ROS may contribute significantly to the modulation of the molecular crosstalk between these cell adhesion receptors, thus opening a novel research avenue.

Furthermore, as the impairment of the integrin-cadherin crosstalk is involved in the development of serious pathological processes, including abnormal angiogenesis, tumor invasion, and metastasis, strategies aimed at controlling ROS homeostasis to preserve the coordinated adhesive and signaling functions of integrins and cadherins might harbor important therapeutic potential for human health.
